# Unravelling the relative roles of top‐down and bottom‐up forces driving population change in an oceanic predator

**DOI:** 10.1002/ecy.1452

**Published:** 2016-08-01

**Authors:** C. Horswill, N. Ratcliffe, J. A. Green, R. A. Phillips, P. N. Trathan, J. Matthiopoulos

**Affiliations:** ^1^ British Antarctic SurveyHigh CrossCambridgeCB3 0ETUnited Kingdom; ^2^ Institute of BiodiversityAnimal Health and Comparative MedicineUniversity of GlasgowGlasgowG12 8QQUnited Kingdom; ^3^ School of Environmental SciencesUniversity of LiverpoolLiverpoolL69 3GPUnited Kingdom

**Keywords:** density dependence, El Niño Southern Oscillation, Macaroni Penguin (*Eudyptes chrysolophus*), Monte Carlo Markov chain, population model, predation, sea surface temperature, seabird, stochastic variable selection

## Abstract

In the open ocean ecosystem, climate and anthropogenic changes have driven biological change at both ends of the food chain. Understanding how the population dynamics of pelagic predators are simultaneously influenced by nutrient‐driven processes acting from the “bottom‐up” and predator‐driven processes acting from the “top‐down” is therefore considered an urgent task. Using a state‐space demographic model, we evaluated the population trajectory of an oceanic predator, the Macaroni Penguin (*Eudyptes chrysolophus*), and numerically assessed the relative importance of bottom‐up and top‐down drivers acting through different demographic rates. The population trajectory was considerably more sensitive to changes in top‐down control of survival compared to bottom‐up control of survival or productivity. This study integrates a unique set of demographic and covariate data and highlights the benefits of using a single estimation framework to examine the links between covariates, demographic rates and population dynamics.

## Introduction

The predator‐driven or “top‐down” view of population control appears to be widely accepted by researchers considering terrestrial (Hairston et al. 1960), freshwater (Carpenter et al. 1985), and intertidal ecosystems (Paine 1980). In contrast, with the exception of human exploitation and fisheries by‐catch, the majority of population change in the pelagic zone is thought to be nutrient driven, or controlled from the “bottom‐up” (Aebischer et al. 1990, Stenseth et al. 2002). This is because the overall structure and functioning of the pelagic system is dominated by physical processes and nutrient fluxes (Pace et al. 1999, Behrenfeld et al. 2006). However, a number of studies have linked population change in pelagic predators to marked adjustments in community structure at lower trophic levels, indicative of top‐down control (e.g., Estes and Duggins 1995, Bascompte et al. 2005, Frank et al. 2005, Springer and van Vliet 2014).

Marine ecosystems are receiving growing attention because anthropogenic drivers have precipitated biological change at both ends of the food chain. For example, in the Antarctic system, rapid regional warming has had a major impact on lower trophic levels (Vaughan et al. 2003, Atkinson et al. 2004), and the composition of predators occupying the upper trophic level has been repeatedly changed by “boom and bust” sealing, whaling, and fishing industries (Agnew 2004). Reliably predicting the population response to future climate change and regulating exploitation pressures at sustainable levels requires certainty in the ecological processes that influence population dynamics. Therefore, unravelling the effects of bottom‐up and top‐down forcing in this region, particularly on species that utilize the open ocean, is considered an urgent task (Smetacek and Nicol 2005). However, reliably separating these effects requires diverse demographic and covariate data that are difficult to collect for ocean‐scale populations. Consequently, studies comparing the relative importance of these population drivers are largely lacking for this system.

The early view was that Antarctic and Sub‐Antarctic oceanic systems were relatively simple, characterized by a large prey resource, principally Antarctic krill (*Euphausia superba*), supporting an assemblage of apex predators (Laws 1977). However, an increasing number of studies have demonstrated that the demographic processes of particular Antarctic predators are controlled by trophic levels both above and below them (Schwarz et al. 2013, Horswill et al. 2016). By considering specific species of seals and seabirds as occupants of intermediate trophic levels, we improve our ability to evaluate the drivers of their population dynamics. This is best assessed using integrated population models (Francis and Sagar 2011, Matthiopoulos et al. 2013, Maunder and Punt 2013, Thomson et al. 2015, Tuck et al. 2015). In particular, Bayesian state‐space approaches permit relationships between covariates and demographic processes to reflect the available knowledge of the system (Buckland et al. 2004). Furthermore, missing data can be imputed and the measurement errors inherent in ecological data can be accounted for by estimation and prediction uncertainty (Buckland et al. 2004).

**Figure 1:**
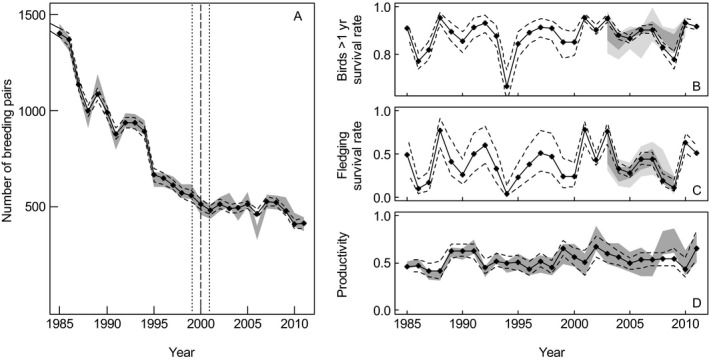
The population dynamics and demography of Macaroni Penguins at Bird Island, South Georgia between 1985 and 2011. (A) The population trajectory. The observed trajectory is shown with confidence interval estimated from the repeated colony counts in gray shading. The approximate point where the gradient of the population trajectory changed (±SE) is shown with a vertical dashed line. In all panels. the median posterior estimates are shown (solid points) with 95% credible interval (dashed line). (B–C) Posterior estimates of survival rates for (B) birds >1 yr old and (C) fledglings. The 95% confidence intervals of the independent survival estimates from the capture–mark–recapture fully time‐dependent model, are shown as the light gray shaded band. For comparison purposes, the survival estimates from the covariate model (Horswill et al. 2014) are also illustrated by the dark gray band. (D) Time series of posterior estimates of productivity rates (scaled to reflect number of chicks per pair). Observed productivity estimates are shaded in gray with confidence intervals taken from the repeated colony counts.

Many populations of Antarctic and Sub‐Antarctic marine predators declined between the 1980s and early 2000s (Reid and Croxall 2001, Woehler et al. 2001, Lyver et al. 2014). For example, Macaroni Penguin (*Eudyptes chrysolophus*) colonies on South Georgia had a net decline of approximately 70% during this time (Trathan et al. 2012; Fig. 1A). Several studies have attributed this to a single driver, including climate (Forcada and Trathan 2009), or elevated levels of competition from the recovering populations of Antarctic fur seals (*Arctocephalus gazella*; Trathan et al. 2012). However, the relative importance of these different drivers is not understood. Furthermore, a recent examination of Macaroni Penguin survival rates highlighted that top‐down control mechanisms should also be considered when examining the demography of this species (Horswill et al. 2014).

In this study, we used an age‐structured state‐space model to integrate a unique set of demographic and covariate data and examine the links between individual covariates, demographic rates, and the overall population trajectory for Macaroni Penguins. This included 28 yr of abundance and productivity data, 8 yr of survival data and a diverse set of covariates. By subjecting this model to a sensitivity analysis, we also estimated the relative contributions of top‐down and bottom‐up forces in generating year‐to‐year variation in demography and population dynamics.

## Methods

### Study system

This study uses data collected from the Fairy Point Macaroni Penguin colony on Bird Island, South Georgia (54°00′ S, 38°03′ W). Abundance and productivity were monitored annually between 1985 and 2012 (Table 1); the total number of breeding pairs was counted at the start of the incubation phase (29 November–10 December), and the number of chicks was counted shortly before fledging (16 February). Productivity is defined as the proportion of breeding pairs that reared a chick to the fledgling count date. Each annual count was repeated at least three times in the field, or to within 10% of the other estimates (methods are further detailed in CCAMLR 2004). Survival data were obtained from a mark–recapture study conducted between 2003 and 2012 based on passive integrated transponders (PIT; Horswill et al. 2014). To remove any temporal correlation introduced by the covariates that were retained in this earlier study, the estimates of survival utilized in the current study were reestimated from a model without covariates, that incorporated age and time‐specific variation and year as a nominal factor variable (recapture and transition rates were specified in agreement with the best candidate model reported by Horswill et al. 2014). This maximally flexible model produced year‐specific estimates of survival with associated yearly measures of uncertainty.

**Table 1:** The candidate covariates used to resolve the population trajectory of Macaroni Penguins at Bird Island, South Georgia

Process and covariate	Data availability	Reference of effect
Survival (fledging)		
Predation pressure	2001–2012	Horswill et al. (2014)
Sea surface temperature anomalies (SSTa)	1985–2012	Horswill et al. (2014)
Survival (>1 yr)		
Predation pressure	2001–2012	Horswill et al. (2014)
SSTa	1985–2012	Horswill et al. (2014)
Productivity		
Female arrival mass	1989–2012	Crawford et al. (2006)
Predation	2001–2012	Le Bohec et al. (2003)
SSTa	1985–2012	Chambers (2004)
Southern Annular Mode (SAM)	1985–2012	Forcada and Trathan (2009)
El Niño/Southern Oscillation (ENSO)	1985–2012	Chambers (2004)
Intraspecific competition	1985–2012	Baylis et al. (2013)
Interspecific competition	1985–2012	Trathan et al. (2012)

*Note:*

The lengths of available time series are also illustrated in Appendix S3.

### State‐space population model

Simultaneous estimation of parameters and hidden states was carried out alongside model selection in a state‐space population model that included (1) coefficients describing the relationship between different covariates and demographic processes between 1985 and 2012 (Appendix S1), (2) Bernoulli selection coefficients that determined the inclusion of each covariate, (3) missing (and thus imputed) segments of demographic and covariate time series, and (4) the magnitude and direction of different observation biases and imprecision (code listed in the accompanying Data S1 file). Inference was undertaken in OpenBUGS (*available online*).^5^ Models were fitted by running three Monte Carlo Markov chains (MCMC) for 1 × 10^6^ iterations and retaining every 100th step in order to increase the effective MCMC sample size for the same amount of computer memory. The first 5,000 MCMC draws were removed as burn‐in. Each chain was initialized at different points in parameter space, and mixing and convergence of the MCMC was examined for each parameter and state. The amount of mixing between the three chains indicated the ability of the model to reach a steady state distribution. Convergence of the chains was confirmed using the Brooks‐Gelman‐Rubin diagnostic tool in the OpenBUGS software (all values <1.02). The final model structure was validated by removing the demographic data and simulating the population time series using the influential covariates and the marginal posterior distributions for the respective parameters. This procedure demonstrated whether the observed population time series could be recreated from the functional structure, the parameter estimates and the covariate data, and by not including the implicit covariance embedded in the MCMC samples was considered a stringent method for testing the descriptive power of the variables (Appendix S2).

#### Covariates

The demographic function of survival included covariates that were demonstrated to be influential at the population level in the analysis of deviance study reported by Horswill et al. (2014); i.e., predation pressure and local sea surface temperature anomalies (SSTa; Eqs. (2), (3); Table 1). Wider testing of candidate covariates in the survival function reduced model convergence and was therefore avoided. For the demographic function of productivity, we selected candidate covariates from information across Spheniscidae (Table 1). Top‐down control was assessed using a proxy of predation pressure. Bottom‐up control was assessed using several proxies of prey availability; female body mass at the start of the breeding season, two measures of competition, as well as one local and two quasi‐remote climatic variables: (1) SSTa, (2) the El Niño/Southern Oscillation (ENSO) phenomenon, and (3) the Southern Annular Mode (SAM).

For the index of predation pressure we used the number of Northern (*Macronectes halli*) and Southern (*M. giganteus*) Giant Petrel chicks successfully reared to fledging. Northern and Southern Giant Petrels are large birds that are predators and scavengers. On Bird Island, these species breed sympatrically at densities that, at the time of publication, were among the highest in the world. Nesting pairs of Giant Petrels in three study areas close to the penguin study colony were visited weekly during incubation and chick‐rearing to determine the local productivity. This measure represents the number of adult Giant Petrels that will be foraging under highly constrained central‐place‐constraint for the whole of the penguin breeding season, including when the Macaroni Penguin chicks fledge. Penguins form a major component of Giant Petrel diet during the breeding season (Bonner and Hunter 1982, Hunter and Brooke 1992), and at Bird Island, this is thought to consist predominantly of adult Macaroni Penguins (Hunter 1983) scavenged from predation events by sub‐adult Antarctic fur seals (Bonner and Hunter 1982). More recently, anecdotal accounts from Bird Island report Macaroni Penguin chicks also being heavily predated by Giant Petrels as they fledge (J. A. Green, P. N. Trathan, and R. A. Phillips, *personal observation*), as well as an adult Macaroni Penguin being killed by a Giant Petrel in waters close to the study colony (J. Gillham, *personal communication*). Therefore, the predation pressure index is taken to reflect predation by both Giant Petrels and sub‐adult Antarctic fur seals.

To examine the influence of Macaroni Penguin body condition on productivity rates we included the body mass of adult females at the start of the breeding season. The Macaroni Penguin breeding season is highly synchronous, and birds were weighed annually between 8 and 9 November using a spring balance ([*n* = 49–59]; ±0.05 kg; Pesola, Baar, Switzerland). A study of Macaroni Penguins breeding in the Indian Ocean did not identify a significant relationship between arrival mass and productivity, however, a positive correlation has been reported in other crested penguins (Rockhopper Penguins *E. chrysocome filholi*; Crawford et al. 2006). The influence of competition during the breeding season was examined using proxies of inter‐ and intraspecific effects. The foraging grounds and diets of breeding Macaroni Penguins and Antarctic fur seals overlap extensively during the breeding season (Reid et al. 1996, Trathan et al. 2006, Staniland et al. 2012). We used the number of Antarctic fur seal pups born on the fur seal study beach at Bird Island (minus those found dead) to provide a measure of female seals foraging within a restricted foraging range during the Macaroni Penguin breeding season. To examine intraspecific competition we used the number of Macaroni Penguin breeding pairs at the study colony. The trajectory of the study colony mirrored other, much larger colonies in the same region (Trathan et al. 2012). Therefore, this measure was taken to reflect the wider population density of penguins.

SSTa are considered to have a major influence on prey biomass within the South Georgia continental shelf zone (Trathan et al. 2003). To reflect local conditions, we used SSTa in the foraging area used by Macaroni Penguins during the breeding season (35.5° W to 44.5° W, 52.5° S to 54.5° S). These data were obtained from the National Oceanographic and Atmospheric Administration (NOAA) International Research Institute (data *available online)* 6. Ocean‐scale climate effects, such as the El Niño Southern Oscillation (ENSO) phenomenon and the Southern Annular Mode (SAM), are associated with major changes in upwelling, SSTa, circumpolar winds (Meredith et al. 2008) and local prey availability (Murphy et al. 2007). These data were obtained from the NOAA Climate Diagnostics Center (data *available online*),^7^ and the NOAA Climate Prediction Center (data *available online*).^8^ Candidate temporal lags for these climatic variables were calculated by summing plausible physical and biological process lags (following Horswill et al. 2014; Appendix S2). El Niño events are associated with warmer temperatures in the Scotia Sea region after approximately a 2‐yr lag, whereas the effects of SAM are typically more immediate, i.e., no lag (Meredith et al. 2008). Biological lags associated with the recruitment of krill to South Georgia were added to the physical lags in two potential spawning and dispersal scenarios. Either spawning occurs across the Scotia Sea with recruitment maintained within that year in all shelf regions (Brierley et al. 1999), or spawning occurs mainly in central and southern areas of the Scotia Sea, with dispersal occurring through interactions with the ocean and sea ice over the next 1–2 yr (Hofmann et al. 1998). All covariates were standardized to have a zero mean and unit variance to promote convergence across parameters with different scales (Congdon 2003). Annual values were calculated following Horswill et al. (2014; Appendix S2).

#### Demographic model

Incomplete attendance histories at the individual level caused by variable recapture rates within seasons precluded the identification of recruitment and missed breeding events. Studies of Crested Penguins marked with flipper bands report that recruitment can occur within the same year as first return following deferred reproduction (+1 yr; Guinard et al. 1998). The mean age of recruitment was therefore taken to correspond with the maximum age of first return for this population (4 yr; Horswill et al. 2014). In the absence of evidence for a significant sex difference in survival rates (Horswill 2015) we assumed a 1:1 sex ratio in the population. To account for the production of one chick per two adults, the model was based on female numbers only, i.e., the number of breeding females (equal to the number of breeding pairs), and the number of female chicks fledged (equal to half the total productivity). The model assumed that all birds aged four and above will breed annually. Although Macaroni Penguins may skip an individual breeding attempt following particularly adverse winter conditions (Williams and Rodwell 1992, Crawford et al. 2006), the study population did not fluctuate widely between years, and the PIT reader used in this study achieved very high recapture rates when it was fully operational (99%; Horswill et al. 2014). Consequently, it appears that intermittent breeding was not a common source of variation during the study period.

The deterministic transition matrix was based on breeding success (*b*_t_) and five state variables: one fledgling (from fledging to 1 yr of age; $\phi_{f,t}$), three sub‐adult (age classes 2–4 have the same survival probability as an adult), and one adult stage ($\phi_{a,t}$):

(1) $$R_{t}=\left(\begin{array}{cccccc} 0 & 0 & 0 & 0 & 0 & \phi_{a,t}\frac{b_{t}}{2}\\ \phi_{f,t} & 0 & 0 & 0 & 0 & 0\\ 0 & \phi_{a,t} & 0 & 0 & 0 & 0\\ 0 & 0 & \phi_{a,t} & 0 & 0 & 0\\ 0 & 0 & 0 & \phi_{a,t} & 0 & 0\\ 0 & 0 & 0 & 0 & \phi_{a,t} & \phi_{a,t} \end{array}\right).$$

Macaroni Penguins lay two eggs, but near‐complete failure of the first‐laid egg means that they effectively produce a single‐egg clutch (Williams 1995). This permitted individual productivity and survival events to be modelled using binomial demographic stochasticity, and the probability of success for each time step using a logit function that incorporated candidate covariates (following Matthiopoulos et al. 2013). For purposes of model selection, a switch variable with an independent Bernoulli 0/1 indicator was used to determine whether each covariate was allowed to operate within the model for any given parameterization (George and McCulloch 1993). By using a uniform prior bounded between 0 and 1 to estimate the parameter of this indicator, we were also able to estimate the probability of inclusion for each covariate (Lunn et al. 2012; Eqs (2), (3), (4)). All prior distributions are detailed further in Appendices S1–S2.

The logit functions for adult and fledgling survival rates are shown in Eqs. (2) and (3). The age‐specific drivers included changing combinations of top‐down control from predation pressure (*P*) and bottom‐up control from SSTa (with a year lag, *S*_*t*−1_):

(2) $$\text{logit}\left(\phi_{f,t}\right)=\alpha_{f,0}+\left(I_{1}\alpha_{1}+I_{2}\alpha_{2}\right)P_{t}+I_{3}\alpha_{3}S_{t-1},$$

(3) $$\text{logit}\left(\phi_{a,t}\right)=\alpha_{a,0}+\left(I_{1}\alpha_{1}\right)P_{t}+I_{3}\alpha_{3}S_{t-1}.$$

Here, *f* and *a* denote birds in the fledging year and birds that are older than 1 yr, respectively. Age dependence was determined by using different parameters for the baseline survival of each age class (α_*f*,0_, α_*a*,0_). Following Horswill et al. (2014) the parameters for the predation (α_1_) and SSTa terms (α_3_) replicated the effect of these covariates across both age classes, and an extra coefficient (α_2_) was included in the function for fledgling survival to allow for an additional component of predation on fledglings (Horswill et al. 2014).

The productivity function (Eq. (4)) included female arrival mass (*A*), environmental forces (SSTa *S*, SAM *M,* and ENSO *E*), inter‐ (*D*) and intraspecific competition (*C*), and predation pressure (*P*):

(4) $$\begin{array}{cc} \text{logit}(b_{t})= & \beta_{0}+I_{1}\beta_{1}A_{t}+I_{2}\beta_{2}P+I_{3}\beta_{3}S_{t}+I_{4}\beta_{4}S_{t-1}\\ & +I_{5}\beta_{5}M_{t}+I_{6}\beta_{6}M_{t-1}+I_{7}\beta_{7}E_{-2}+I_{8}\beta_{8}E_{-3}\\ & +I_{9}\beta_{9}D_{t}+I_{10}\beta_{10}C_{t} \end{array}.$$

The number of breeding females estimated by the process model was fed back into the productivity function as the auto‐covariate *C*.

#### Observation model

Observation error was stochastically assigned in each time step to account for annual variation associated with detection and unspecified covariates. For survival, the maximum range of uncertainty was set using the 95% confidence intervals (CIs) from the mark–recapture analysis in order to encompass both process variability and observation error (Fig. 1B‐C). The true range of residuals for survival within the state‐space model will be narrower because some variability will be accounted for by the inclusion of covariates. Survival estimates were assumed to have a normal error distribution where the mean was equal to the mark‐recapture estimate, and the variance was set from a uniform distribution. Here, the maximum value was stochastically assigned from a gamma distribution that represented the available range of 95% CIs. For productivity, observation error was also applied through a normal error structure on the number of breeding females and the number of female chicks. The mean was equal to the colony count estimate and the variance was derived from the range of the repeated counts. These data were available for 5 yr between 2007 and 2012; therefore the same variance was applied to all years. The variance (*s* ^2^) was calculated using the mean variance of the annual counts (${\overset{¯}{\sigma }}^{2}$ = 1,727.3) and the mean number of counts ($\overset{¯}{n}$ = 4.7 repeated counts):

(5) $$s^{2}=\frac{{\overset{¯}{\sigma }}^{2}}{\overset{¯}{n}}=0.0028.$$

As with simpler regression analyses, the covariate data will also contain some observation error. Ignoring these uncertainties in a typical regression framework that uses model selection (e.g., via information criteria) can lead to spurious correlations. It was not computationally feasible to propagate these sources of uncertainty through the state‐space model, however, the embedded model selection approach essentially operates as a Bayesian equivalent to model averaging (Lunn et al. 2012). Therefore, this approach should reduce the probability of spurious correlations (Burnham and Anderson 2002).

#### Missing data

Missing data on female mass at the start of the breeding season were estimated as a normal variate with an expectation equal to the observed data (Table 1; Appendices S2–S3). The missing segment of the predation pressure time series was modelled as a random walk through time to enable serial autocorrelation to be included in the process (Table 1; Appendices S2–S3).

### Variables influencing the population trajectory

To identify a suitable splitting point for presenting summary statistics on the early and later parts of the time series, we fitted a broken‐stick GLM to the raw data using the statistical package segmented in program R (Muggeo 2008). To quantify the relative importance of each covariate in determining the observed population trajectory, we compared the estimated population size between model iterations where the variable selection terms indicated specific covariates as being included. For covariates that were included in all model iterations, we refitted the model with the respective selection term set to 0. Years where the imputed values of missing covariate data may have been overestimated were removed from this analysis to minimize bias. This was assessed by examining the population trajectory for anomalous changes that may result in residual variation being artificially attributed to an imputed covariate value (Appendix S2). Repeated bootstrap sampling (5000 samples) was used to undertake multiple pairwise comparisons of the population trajectories estimated with and without the covariate included, calculating the squared residuals between randomly selected MCMC iterations from each scenario. The covariates were then ranked by these statistics to determine their relative influence on the population trajectory. Higher values and greater variability indicated that excluding a covariate reduced the model's ability to recreate the population size.

## Results

### Model fit

The full model (Fig. 1A) and the validation model that did not include the demographic time series (Appendix S4) both convincingly recreated the observed population trajectory. Therefore, we infer that the population trajectory was adequately described by the parameters and covariates considered.

### Missing covariate data

**Figure 2:**
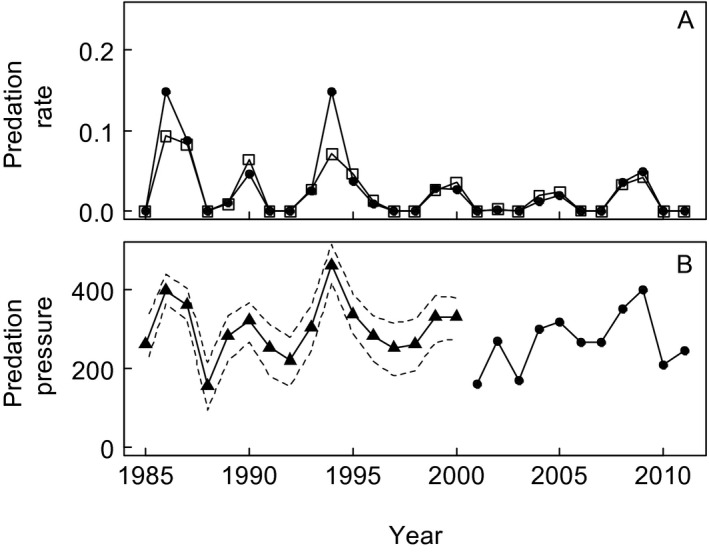
(A) Approximate predation rate of Macaroni Penguins per Giant Petrel based on the total number of Giant Petrels in the study areas (methods detailed in Appendix S2; penguins older than 1 yr, solid circles; fledglings, open squares). (B) The predation pressure index reflecting the productivity rate of northern and southern Giant Petrels at Bird Island, South Georgia. Observed values (i.e., number of chicks fledged from the study area) are shown as solid circles and imputed values are shown as solid triangles with 95% credible interval.

The imputed segment of the predation pressure time series was slightly higher and more variable (de‐standardized ${\overset{¯}{x}}_{<2000}=302,\;{SD}_{<2000}=75$; Fig. 2A) than the segment of observed data (de‐standardized ${\overset{¯}{x}}_{\geq 2000}=269,\;{\text{SD}}_{\geq 2000}=68$; Fig. 2A). This was largely attributed to elevated estimates of predation during 1986, 1987, 1990, and 1994. These values may have been inflated by the concurrent population declines (Figs. 1A and 2B), and therefore were removed from the sensitivity analysis. The imputed values for body mass of females at the start of the breeding season were highly similar to the observed values (de‐standardized imputed values ${\overset{¯}{x}}_{<1989}=4.99,\;{\text{SD}}_{<1989}=0.19$, de‐standardized observed values ${\overset{¯}{x}}_{\geq 1989}=5.04,\;{\text{SD}}_{\geq 1989}=0.16$).

### Variables influencing survival rates

**Figure 3:**
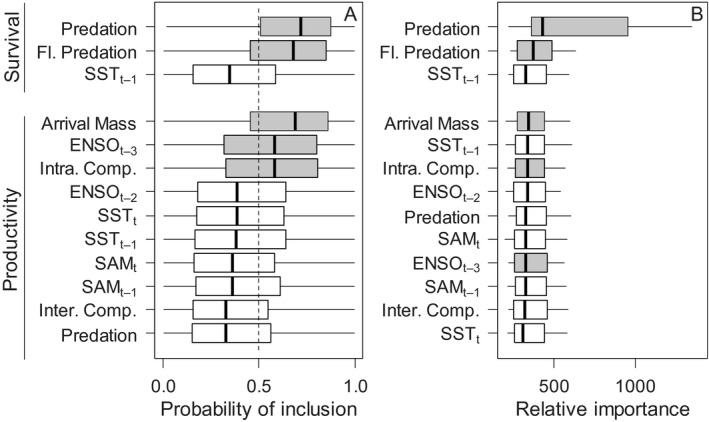
(A) The probability of each covariate influencing the population trajectory estimated using stochastic variable selection. Dashed line at 0.5; a covariate scoring predominantly above this will operate in more than 50% of model parameterizations. (B) The relative importance of each covariate in resolving the population trajectory; higher values and greater variability indicates more influence. Covariates are ordered within each demographic rate by the medianposterior estimate; sea surface temperature anomalies (SSTa); El Niño/Southern Oscillation (ENSO); Southern Annular Mode (SAM); time lags shown in subscript. Variables indicated as being included in the majority of model iterations based on variable selection are shaded gray; those excluded are white (box metrics: central line, median; box, interquartile range; whisker, 1.5 × inter‐quartile range).

The mean survival rate for the fledgling age class was slightly lower during the period of population decline compared to the period of population stability (${\overset{¯}{x}}_{<2000}=0.37,\;{\overset{¯}{x}}_{\geq 2000}=0.43$. In addition, annual variability in fledgling survival was consistently high across both time periods (${\text{SD}}_{<2000}=0.20,\;{\text{SD}}_{\geq 2000}=0.22$; Fig. 1B; Appendix S5). Likewise, mean survival rates in the older age class also increased slightly (${\overset{¯}{x}}_{<2000}=0.87,\;{\overset{¯}{x}}_{\geq 2000}=0.89$), however variability was consistently low (${\text{SD}}_{<2000}=0.07,\;{\text{SD}}_{\geq 2000}=0.05$;Fig. 1C; Appendix S5). Based on variable selection, both of the age specific predation effects were influential (Fig. 3A), and the directional influence followed Horswill et al. (2014; Appendix S1). The probability of inclusion for the main predation term was consistently above 0.5; i.e., this term was included in all model iterations. The effect of SSTa on survival rates was not resolvable from variable selection or posterior credible intervals (Appendix S1; Fig. 3A). Despite this, the estimates of survival from the state‐space model predominantly occurred within the confidence interval of the covariate model that was reported in our earlier study (Horswill et al. 2014), with lower values of survival estimated for the older age class during 2003 and 2004 (Fig. 1B,C).

### Variables influencing productivity rates

Productivity increased during the study period (linear regression: *F* = 5.11, df* *= 25, *P* = 0.03), and values were lower during the period of population decline (${\overset{¯}{x}}_{<2000}=0.51,\;{\overset{¯}{x}}_{\geq 2000}=0.55$; Fig. 1D; Appendix S5). Based on variable selection and posterior credible intervals, annual productivity was positively influenced by body mass of females at the start of the breeding season, and negatively influenced by intraspecific competition and ENSO with a 3‐yr lag (Fig. 3A). The remaining covariates were not resolvable based on variable selection or posterior credible intervals (Fig. 3A).

### Variables influencing the population trajectory

The breeding population declined at 6.5% (SE = 0.6) per year from 1985 until ca. 2000, the trend thereafter could not be reliably resolved; declining at 1.2% (SE = 1.1; Fig. 1A). Prior to 2000, the proportion of a cohort that recruited to the breeding population at Bird Island was 0.06 (SD = 0.04) and the mean rate of adult mortality was 0.13 (SD = 0.07; Appendix S5). After 2000, the rate of recruitment was 0.09 (SD = 0.04) and the rate of adult mortality was 0.11 (SD = 0.05; Appendix S5). The sensitivity analysis was carried out using a 24‐yr time series (Appendix S2), and this population trajectory was most sensitive to the removal of predation pressure from the survival functions, especially the main predation term (Fig. 3B). The other covariates were relatively similar in their effect on population size (Fig. 3B).

## Discussion

Unravelling the relative importance of bottom‐up and top‐down forcing in the pelagic zone is central to regulating fishery pressures at sustainable levels, and predicting how marine systems will be influenced by future climatic changes. We sought to examine the links between individual covariates, demographic rates and the overall population trajectory of Macaroni Penguins, and quantify the relative importance of bottom‐up and top‐down drivers acting through different demographic rates.

### Variables influencing survival rates

Between 1987 and 1990 the survival rate of Macaroni Penguins from Bird Island that were marked with flipper‐bands was estimated to be $\overset{¯}{x}∼0.75$ (σ∼0.06) (Williams and Rodwell 1992). We estimated adult survival for the same period to be higher and infer that flipper‐banding may have impaired survival rates (Saraux et al. 2011) during the Williams and Rodwell (1992) study. An alternative explanation is that survival rates estimated by Williams and Rodwell (1992) were based on visual recapture assuming 100% recapture rates that resulted in underestimation. In contrast, our study incorporated errors associated with incomplete detection within the observation model for survival. In agreement with Horswill et al. (2014), survival rates were generally lower during the fledgling year, and increased from age one. However, under certain conditions (low predation pressure), the survival rates of the two age classes were comparable (Fig. 1B–C).

The estimates of survival during the period of population stability were highly similar to those reported by Horswill et al. (2014) for the same time period. The lower estimates generated by the present study for the older age class during 2008 and 2009 is likely to represent the lack of inference attributed to SSTa. This also demonstrates the relative significance of the predation pressure covariate in resolving this demographic process. A negative relationship between survival and predation pressure agrees with dietary analysis that shows Macaroni Penguins form a major component of Giant Petrel diet during the breeding season (Hunter 1983). However, years with particularly low survival rates for Macaroni Penguins also coincided with a decrease in vital rates amongst other species of marine predators that breed on Bird Island. For example, during 1987, the survival rates of Macaroni and Gentoo Penguins (*Pygoscelis papua*) decreased (Williams and Rodwell 1992), and following the 1987 winter, the breeding season of Gentoo Penguins and Antarctic fur seals was delayed (Duck 1990, Williams 1990). A delay in the onset of breeding may indicate adverse climatic conditions during the preceding winter (e.g., Barbraud and Weimerskirch 2006). In addition, rates of productivity at Bird Island were reduced for several marine predator species during 1994 (Reid and Croxall 2001), and finally, Gentoo Penguins experienced high adult mortality and very low productivity during 2009 (British Antarctic Survey, *unpublished data*). Therefore, the predation pressure index may represent mechanisms acting at the community level, and further work is needed to examine the interaction between bottom‐up and top‐down forces during adverse climatic conditions (e.g., Votier et al. 2004).

### Variables influencing productivity

The mean productivity rate during the period of population decline was similar to other decreasing populations of Macaroni Penguins breeding in colonies of comparable size ($\overset{¯}{x}=0.46-0.57$ chicks per pair; Crawford et al. 2006). However, by the end of the study period, annual rates were comparable to an increasing population of Rockhopper Penguins ($\overset{¯}{x}=0.64$ chicks per pair; Baylis et al. 2013). Observational studies indicate that Giant Petrels can depredate penguin chicks at the colony (Le Bohec et al. 2003), however, this foraging strategy has rarely been observed at Bird Island, where predation of Macaroni Penguin chicks appears to be focused on the fledging period. In agreement with this, top‐down control was not identified as being influential to productivity. Instead, low rates of productivity were consistently associated with a decrease in female body mass at the start of the breeding season and a higher value of ENSO; i.e., an El Niño event. A positive relationship between arrival mass and productivity has not been previously shown for this species, however, it has been reported for other crested penguins (Crawford et al. 2006). Likewise, a negative relationship between productivity and ENSO has been previously documented in other species of penguin (e.g., Chambers 2004). Arrival mass and ENSO are bottom‐up processes representing local prey availability immediately before and during the breeding season (Murphy et al. 2007). During this period the study population of Macaroni Penguins are foraging locally (Horswill et al., 2014), and therefore a change in local food availability seems the most likely explanation for lower productivity rates).

The arrival body mass of female Macaroni Penguins decreased between 1988 and 1992 (Reid and Croxall 2001). Furthermore, the ENSO had a marked preponderance of El Niño compared with La Niña events between 1983 and 2008 (Meredith et al. 2008). Consequently, the overall increase in productivity between 1985 and 2012 cannot be attributed to either of these variables; i.e., the predicted trend based on the observed change in these covariates would have been in the opposite direction to that observed. Productivity rates also demonstrated a negative relationship with intraspecific competition, such that productivity increased at lower population densities. Here, the proposed mechanism is principally a product of resource competition, whereby per capita availability of prey and high quality nesting sites increased as the population declined. A negative relationship between population density and productivity has been previously documented in several species of seabird (e.g., Weimerskirch and Jouventin 1987, Frederiksen and Bregnballe 2001), including penguins (Rockhopper Penguins; Baylis et al. 2013).

### Variables influencing the population trajectory

The colony of Macaroni Penguins examined in this study declined rapidly between 1985 and the early 2000s because recruitment did not sufficiently balance adult mortality rates (Appendix S5). The rate of decline was similar to other much larger colonies in the same region (Trathan et al. 2012), and therefore it seems likely that the mechanisms discussed here were influential to the population more broadly. The model's ability to replicate the change in population trajectory without the observed time‐series of demographic data indicates that the principal contributing mechanisms were captured, namely predation, arrival mass, ENSO and intraspecific competition. In agreement with life history theory on long‐lived species, population size was considerably more sensitive to covariates acting on adult survival rates, compared to those acting on juvenile survival and productivity (Sæther and Bakke 2000). However, the additional component of predation pressure acting on fledgling survival rates appeared to be slightly more influential than bottom‐up control of adult survival rates. This is likely to reflect the lack of influence attributed to SSTa across both survival functions. Therefore, as time‐series of demographic and covariate data are extended, it would be worthwhile evaluating alternative bottom‐up covariates as drivers of survival. Finally, the bottom‐up variables acting through productivity generated a similar population‐level effect to SSTa acting through survival. Consequently, it appears that the population dynamics of Macaroni Penguins are relatively canalized (i.e., preserved) against climate variations.

## Conclusions

This study integrated a unique set of demographic and covariate data in order to assess how an ocean‐scale population is influenced by bottom‐up and top‐down drivers. We conclude that the population of Macaroni Penguins at Bird Island rapidly declined between 1985 and the early 2000s following an imbalance between recruitment to the breeding population and adult mortality. The population later stabilized following an increase in survival and productivity. It was not possible to reliably attribute the increase in survival to a single factor; however, the increase in productivity appeared to be driven by a bottom‐up negative feedback with population size. Despite this, population size was considerably more sensitive to changes in top‐down control of survival rates, compared to bottom‐up control of survival or productivity. Under the observed conditions, we can predict that a continued increase in the population size of Giant Petrels or a shift in their predatory behavior (to include chicks at the colony) could rapidly destabilize the penguin population. More broadly, this study highlights the importance of considering how pelagic predators are influenced by multiple drivers when examining their population dynamics, and assessing options for conservation management.

## Notes

5
http://www.mrc-bsu.cam.ac.uk/bugs/

6
http://iridl.ldeo.columbia.edu/SOURCES/.NOAA/.NCEP/.EMC/.CMB/.GLOBAL/.Reyn_SmithOIv2/.monthly/.SSTa/

7
http://www.cdc.noaa.gov/people/cathy.smith/best/

8
http://www.cpc.ncep.noaa.gov/
