# Supplementary material for: Unravelling the relative roles of top‐down and bottom‐up forces driving population change in an oceanic predator
Source: Ecology. 2016 Aug 1;97(8):1919–28. doi: 10.1002/ecy.1452 (PMC5008121; doi:10.1002/ecy.1452)
Supplement: Supplementary file 1 [file ECY-97-1919-s001.doc]

**Appendix S1 for Horswill et al*.* (2016): Unravelling the relative roles of top-down and bottom-up forces driving population change in an oceanic predator**

## Table S1. Prior and posterior information for the demographic parameters on the linear predictor scale. Parameter notation matches equations 2-4.

| Parameter | | Prior distribution | | Posterior distribution | |
| --- | --- | --- | --- | --- | --- |
|  |  | Median | 95-percentiles | Median | 95-percentiles |
| $\alpha_{f,0}$ | First year survival in 1985 | 0.50 | (0.00, 1.00) | 0.38 | (0.44,0.52) |
| $\alpha_{a,0}$ | Adult survival in 1985 | 0.50 | (0.00, 1.00) | 0.89 | (0.89,0.92) |
| $\alpha_{1}$ | First year component of predation | -0.50 | (-1.00, 0.00) | -0.49 | (-0.83, 4.51 x10^-4^) |
| $\alpha{}_{2}$ | Predation pressure (giant petrel productivity) | -0.50 | (-1.00, 0.00) | -0.52 | (-0.71,  -0.40) |
| $\alpha_{3}$ | SSTa_-1_ | 0.00 | (-1.00, 1.00) | -1.80x10^-4^ | (-0.20, 0.05) |
| $\beta_{0}$ | Productivity in 1985 | 0.50 | (0.00, 1.00) | -0.77 | (-1.09,  -0.45) |
| $\beta_{1}$ | Female arrival mass | 0.00 | (-1.00, 1.00) | 0.42 | (-3.10 x10^-4^, 0.64) |
| $\beta_{2}$ | Predation pressure (giant petrel productivity) | 0.00 | (-1.00, 1.00) | 1.41 x10^-4^ | (-2.14 x10^-3^, 0.10) |
| $\beta_{3}$ | SSTa_0_ | 0.00 | (-1.00, 1.00) | 3.95 x10^-4^ | (-2.28 x10^-3^, 0.30) |
| $\beta_{4}$ | SSTa_-1_ | 0.00 | (-1.00, 1.00) | -3.93x10^-4^ | (-0.18,  2.44 x10^-3^) |
| $\beta_{5}$ | SAM_0_ | 0.00 | (-1.00, 1.00) | 2.09x10^-4^ | (-2.47x10^-3^, 0.20) |
| $\beta_{6}$ | SAM_-1_ | 0.00 | (-1.00, 1.00) | -1.72x10^-4^ | (-0.20, 0.03) |
| $\beta_{7}$ | ENSO_-2_ | 0.00 | (-1.00, 1.00) | -4.30 x10^-4^ | (-0.16,  1.99x10^-3^) |
| $\beta_{8}$ | ENSO_-3_ | 0.00 | (-1.00, 1.00) | -0.09 | (-0.20,  1.52 x10^-3^) |
| $\beta_{9}$ | Inter-specific competition (fur seal productivity) | 0.00 | (-1.00, 1.00) | 6.16x10^-5^ | (-2.57x10^-3^, 0.08) |
| $\beta_{10}$ | Intra-specific competition (penguin population size) | 0.00 | (-1.00x10^-3^, 1.00x10^-3^) | -4.38x10^-4^ | (-1.47x10^-3^, 1.46x10^-3^) |

Notation: Sea surface temperature anomalies (SSTa); Southern Annular Mode (SAM); El Niño/Southern Oscillation (ENSO); time lags given in subscript.

## 
