# Supplementary material for: Unravelling the relative roles of top‐down and bottom‐up forces driving population change in an oceanic predator
Source: Ecology. 2016 Aug 1;97(8):1919–28. doi: 10.1002/ecy.1452 (PMC5008121; doi:10.1002/ecy.1452)
Supplement: Supplementary file 2 [file ECY-97-1919-s002.doc]

**Appendix S2 for Horswill et al*.* (2016): Unravelling the relative roles of top-down and bottom-up forces driving population change in an oceanic predator**

## Detailed methods

Model validation

The final model was validated by simulating the population trajectory without the demographic data or the covariates indicated as being insignificant based on variable selection. Therefore, the simulation was based on demographic functions, the covariates selected as influential by the full model and the parameter estimates. The parameters and initial population structure were assigned as normal variates with an expectation and variance equal to the marginal posterior distributions derived from the full model (Appendix S1). This process highlighted whether the model fit was dependent on the inclusion of the demographic data.

*Candidate covariates*

Table S1. Temporal resolution of climatic covariates.

| Covariate | Study period | Time  lag (years) | Parameter |
| --- | --- | --- | --- |
| Local sea surface temperature anomalies (SSTa) | Annual (October *t-1* to September *t*)  Annual (October *t-2* to September *t-1*) | 0  1 | SST_t_  SST_t-1_ |
| El Niño/Southern Oscillation Index (ENSO) | Summer (October *t-3* to March *t-2*)  Summer (October *t-4* to March *t-3*) | 2  3 | ENSO_t-2_  ENSO_t-3_ |
| Southern Annular Mode index (SAM) | Summer (October *t-1* to March *t*)  Summer (October *t-2* to March *t-1*) | 0  1 | SAM_t_  SAM_t-1_ |

*Demographic model*

Variable selection priors were used to numerically evaluate the probability of inclusion for each covariate (Lunn et al. 2012). For purposes of model selection, a switch variable with an independent Bernoulli 0/1 indicator was used to determine whether a specific covariate was allowed to operate within the model for any given parameterisation (George and McCulloch 1993). The effect of the parameter was then assigned a “spike and slab” prior (Mitchell and Beauchamp 1988), that was concentrated around zero when the term was excluded and drawn from a uniform distribution when the variable was selected (Data File S1, Observation model). The uniform distribution for a selected covariate was bounded between -1 and 1 to enable the direction of the respective parameters to be informed through the integrated analysis. To support convergence, the predation parameters in the survival function were given negative prior distributions in line with the published information on these effects; i.e., within a given year the survival rate of macaroni penguins had a negative relationship with predation pressure (Horswill et al. 2014); $\nu_{j}[2]\text{\textasciitilde dunif(-1,0)}$. The intercept terms ($\alpha_{f,0},\alpha_{a,0},\beta_{0}$) were given uniform priors bounded between 0 and 1 to enable meaningful probabilities to be estimated; *dunif* ~ (0,1). The auto-covariate ($C$) was generated as a state-variable in the model so it could not be standardised within the model framework. Because this covariate was on a much larger scale than the standardised covariates, the variance of the respective prior was scaled accordingly (Appendix S1).

The number of breeding females in the starting year was estimated from the mean count of breeding pairs in 1985 under a uniform prior. Here, the range was informed by the potential error associated with the 1985 colony count. This was estimated by comparing the mean colony count with a corresponding photo of the entire colony (n=5 years). Photo counts were repeated by 5 persons, and the average measurement error was estimated by subtracting the mean photo count from the mean colony count for each available year (error = ±30 birds). The total number of females in each of the 4 age-states was modelled from this stochastic estimate assuming a stable age structure with mean demographic rates equal to the observed time-series (Leslie 1945). This allowed the population trajectory in 1985 to decline at 4%.

*Missing data*

A normal distribution was used to impute the missing values of female body mass at the start of the breeding season. This structure enabled the predicted values to take positive or negative values, in line with the standardised time series of covariate data. The missing segment of the predation pressure time series was modelled as a random walk through time to allow serial autocorrelation to be included in the process. Here, data points are estimated as normal variates with an expectation equal to the previous year (observed or estimated,$P_{t}$). The precision () between sequential data points was given a uniform prior distribution between zero and the maximum difference that was observed between sequential data points:

$\begin{matrix} P_{t+1}\sim Normal(P_{t},\tau) \\ \tau\sim Uniform(1.51,10\text{x}10^{5}) \end{matrix}$ (eqn. S1)

To verify that this precision could be considered representative of the entire study period, the sequential difference was extrapolated according to recent full island censuses ($\bar{\text{x}}=329.2,SD=261.1$; BAS, unpublished data) and compared to productivity data collected for the whole island between 1978 and 1982 ($\bar{\text{x}}=331.8,SD=240.1$; Hunter, 1984). The first value of predation ($P_{1985}$) was assigned a uniform prior with a range equal to the observed data. The tails of the normal distribution in the random-walk model permit estimated values of predation pressure to become negative. Therefore the likelihood of this state was truncated at the standardised value of zero predation pressure.

*Model sensitivity to covariates*

To highlight years where the imputed missing values of predation pressure may have been overestimated due to a lack of signal in the other covariates we quantified the number of deaths attributed to predation (Fig. 2, Main text). This was estimated by simultaneously modelling four penguin survival functions: two that included the predation terms in the functional equation (eqn. 2-3, main text), and two that did not (eqn. S2-S3). The difference in survival between and for the fledgling age class, and and for the older age class, was converted into a predation rate by multiplying the number of individuals by the number of penguins in each age class and dividing by the number of giant petrels present (i.e. 3x the predation pressure index; the breeding pair and the chick). The sensitivity analysis was carried out using a 24 year time series.

(eqn. S2)

(eqn. S3)

**References**

George, E. I., and R. E. McCulloch. 1993. Variable selection via Gibbs sampling. Journal of the American Statistical Association 88:881–889.

Horswill, C., J. Matthiopoulos, J. A. Green, M. P. Meredith, J. Forcada, H. J. Peat, M. Preston, P. N. Trathan, and N. Ratcliffe. 2014. Survival in macaroni penguins and the relative importance of different drivers: individual traits, predation pressure and environmental variability. Journal of Animal Ecology 83:1057–1067.

Hunter, S. 1984. Breeding biology and population dynamics of giant petrels Macronectes at South Georgia (Aves: Procellariiformes). Journal of Zoology 203:441–460.

Leslie, P. H. 1945. On the use of matrices in certain population mathematics. Biometrika 33:183–212.

Lunn, D., C. Jackson, N. Best, A. Thomas, and D. Spiegelhalter. 2012. The BUGS Book: A Practical Introduction to Bayesian Analysis. Chapman & Hall/CRC Texts in Statistical Science, Boca Raton.

Mitchell, T., and J. Beauchamp. 1988. Bayesian variable selection in linear regression. Journal of the American Statistical Association 83:1023–32.
