# Supplementary material for: Unravelling the relative roles of top‐down and bottom‐up forces driving population change in an oceanic predator
Source: Ecology. 2016 Aug 1;97(8):1919–28. doi: 10.1002/ecy.1452 (PMC5008121; doi:10.1002/ecy.1452)
Supplement: Supplementary file 3 [file ECY-97-1919-s003.doc]

**Appendix S3 for Horswill et al*.* (2016): Unravelling the relative roles of top-down and bottom-up forces driving population change in an oceanic predator**

Figure S1. Time series of available covariate data. Notation: Sea surface temperature anomalies (SSTa); Southern Annular Mode (SAM); El Niño/Southern Oscillation (ENSO); time lags given in subscript.
