# Supplementary material for: Unravelling the relative roles of top‐down and bottom‐up forces driving population change in an oceanic predator
Source: Ecology. 2016 Aug 1;97(8):1919–28. doi: 10.1002/ecy.1452 (PMC5008121; doi:10.1002/ecy.1452)
Supplement: Supplementary file 4 [file ECY-97-1919-s004.doc]

**Appendix S4 for Horswill et al*.* (2016): Unravelling the relative roles of top-down and bottom-up forces driving population change in an oceanic predator**

## Figure S1. The result of the model validation exercise. The population trajectory of macaroni penguins at Bird Island, South Georgia, was simulated as a function of the parameter estimates and the covariates. Posterior median values (circles) and credible interval (dashed lines) are shown against the observed population trajectory with confidence interval estimated from the repeated counts (shaded grey).

## 
