# Supplementary material for: Unravelling the relative roles of top‐down and bottom‐up forces driving population change in an oceanic predator
Source: Ecology. 2016 Aug 1;97(8):1919–28. doi: 10.1002/ecy.1452 (PMC5008121; doi:10.1002/ecy.1452)
Supplement: Supplementary file 5 [file ECY-97-1919-s005.doc]

**Appendix S5 for** **Horswill et al*.* (2016): Unravelling the relative roles of top-down and bottom-up forces driving population change in an oceanic predator**

## Table S1. Mean and standard deviation of demographic rates before and after the change in macaroni penguin population trajectory that occurred *c.* 2000.

|  | | 1985-1999 | 2000-2012 |
| --- | --- | --- | --- |
| Survival (Fledging year) | | 0.37 ±0.20 | 0.43 ±0.22 |
| Survival (>1 year) | | 0.87 ±0.07 | 0.89 ±0.05 |
| Recruitment | | 0.06 ±0.04 | 0.09 ±0.04 |
| Productivity | Female chicks female^-1^ | 0.26 ±0.04 | 0.28 ±0.03 |
|  | Chicks pair^-1^ | 0.51 ±0.08 | 0.55 ±0.06 |
