# Supplementary material for: Unravelling the relative roles of top‐down and bottom‐up forces driving population change in an oceanic predator
Source: Ecology. 2016 Aug 1;97(8):1919–28. doi: 10.1002/ecy.1452 (PMC5008121; doi:10.1002/ecy.1452)
Supplement: Supplementary file 7 [file ECY-97-1919-s007.docx]

# C. Horswill, N. Ratcliffe, J. A. Green, R.A. Phillips, P.N. Trathan & J. Matthiopoulos. 2016. Unravelling the relative roles of top-down and bottom-up forces driving population change in an oceanic predator. *Ecology*

### Supplement

OpenBUGS code for population model

### Author(s)

# Cat Horswill^*1,2,3^, Norman Ratcliffe^1^, Jonathan A. Green^4^, Richard A. Phillips^1^, Phil N. Trathan^1^ & Jason Matthiopoulos^2^

^1^British Antarctic Survey, High Cross, Cambridge, CB3 0ET

^2^Institute of Biodiversity, Animal Health & Comparative Medicine, University of Glasgow, G12 8QQ

^3^*Current address:* British Trust for Ornithology, The Nunnery, Thetford, Norfolk, IP24 2PU

^4^School of Environmental Sciences, University of Liverpool, L69 3GP

### *Corresponding author:* Cat Horswill, British Trust for Ornithology, The Nunnery, Thetford, Norfolk, IP24 2PU. E-mail: [catrsw@gmail.com](mailto:catrsw@gmail.com)

###

### File list in DataS1.zip

Horswill_Data_S1.txt

**Description**

This script was written in OpenBUGS. It runs a state-space integrated population model to examine the links between covariates, demographic rates and the population dynamics of macaroni penguins. Variable selection priors are used to numerically evaluate the probability of inclusion for each covariate. The macaroni penguin data were collected at the Fairy Point colony on Bird Island, South Georgia.
